# Ion Beam-Induced Luminescence (IBIL) for Studying Manufacturing Conditions in Ceramics: An Application to Ceramic Body Tiles

**DOI:** 10.3390/ma17205075

**Published:** 2024-10-17

**Authors:** Victoria Corregidor, José Luis Ruvalcaba-Sil, Maria Isabel Prudêncio, Maria Isabel Dias, Luís C. Alves

**Affiliations:** 1 Centro de Ciências e Tecnologias Nucleares, Instituto Superior Técnico, Campus Tecnológico e Nuclear, Universidade de Lisboa, E.N. 10 (km 139.7), 2695-066 Bobadela LRS, Portugal; iprudenc@ctn.tecnico.ulisboa.pt (M.I.P.); isadias@ctn.tecnico.ulisboa.pt (M.I.D.); lcalves@ctn.tecnico.ulisboa.pt (L.C.A.); 2 Instituto de Física, Universidad Nacional Autónoma de Mexico, Mexico City 04510, Mexico; sil@fisica.unam.mx

**Keywords:** luminescence, amorphous materials, ceramics, photoemission, ionoluminescence, tiles

## Abstract

The first experimental results obtained by the ion beam-induced luminescence technique from the ceramic bodies of ancient tiles are reported in this work. The photon emission from the ceramic bodies is related to the starting minerals and the manufacturing conditions, particularly the firing temperature and cooling processes. Moreover, the results indicate that this non-destructive technique, performed under a helium-rich atmosphere instead of an in-vacuum setup and with acquisition times of only a few seconds, presents a promising alternative to traditional, often destructive, compositional characterisation methods. Additionally, by adding other ion beam-based techniques such as PIXE (Particle-Induced X-ray Emission) and PIGE (Particle-Induced Gamma-ray Emission), compositional information from light elements such as Na can also be inferred, helping to also identify the raw materials used.

## 1. Introduction

Non-destructive and non-invasive characterisation techniques are in high demand, especially when the characterisation is focused on the study of unique or valuable cultural heritage (CH) artefacts. Ion beam analytical (IBA) techniques are among these non-destructive and non-invasive techniques. They involve the use of highly energetic ion beams (usually protons with energies in the order of MeV) which are directed towards the sample under study. Thus, it will emit secondary radiation and particles which, when detected and analysed, can provide valuable information about the sample. Particle-Induced X-ray Emission (PIXE) and Particle-Induced Gamma-ray Emission (PIGE) are good examples of IBA techniques which give information about the elemental composition of a sample with high accuracy and sensitivity (to the μg/g level) [1,2]. Ion beam-induced luminescence (IBIL), also known as ionoluminescence (IL), is another IBA technique based on the analysis of the photons emitted by the sample in the UV-VIS region as a result of outer-shell electron transitions, which provides extra information about the chemical state of the ions or the local chemical bindings in the crystal lattice [3,4,5].

In general, IBA techniques have been applied to study different types of CH materials including metals, stained glass, easel paintings, and manuscripts written with iron gall inks [6,7,8,9,10,11,12,13]. In fact, the development of thin windows with enough mechanical strength to withstand the pressure difference between atmospheric conditions and a vacuum allowed the extraction of ion beams into air, and the performance of IBA measurements under atmospheric conditions. This approach eliminates, for example, limitations related to sampling, such as the size/shape required of the objects in order for them to fit in the vacuum analysis chamber [14,15,16].

Despite the fact that IBIL is a very powerful IBA technique, its applications to cultural heritage have not been fully explored. In the field of archaeometry, most of the experiments using IBIL have been focused on the study of minerals provenance [17,18,19,20,21,22,23] and help to distinguish between natural and artificial gemstones or precious stones such as sapphires and diamonds [24,25]. It has been also applied to the study of inorganic painting pigments [26] and the radiation hardness of white pigments, mostly those which include carbonate-based natural painting pigments, through the formation of colour centres [27].

The ceramic body of tiles acts as support for the glaze layer. It is characterised by a mixture of clay minerals, feldspars, metal oxides, and silica, as well as the potential addition of other components, which is strongly dependent on the local geology [28], particularly in the oldest tiles. During the firing process, the ceramic paste undergoes physical and chemical changes, which depend on the temperature, heating cycles, firing time, and even the atmosphere inside the kiln. These conditions will affect the colour, porosity, hardness, and final composition of the ceramic, among other characteristics [29]. Beyond simple aesthetics, ceramic tiles with dark colours were commonly undesired, whereas the creamy and whiter pastes were preferable as they allow for more vivid surface glaze colours. To achieve this, additives such as calcite were intentionally introduced to the starting mix materials of the paste. During the firing process, calcite decomposes (at temperatures above 800 °C), creating new compounds and incorporating iron ions into the lattice [30]. In this way, the concentration of iron oxides responsible for the red or brown colours are reduced [31], thus decreasing the red hue.

Various techniques are documented in the literature for characterising archaeological ceramics in terms of composition, firing temperature conditions, and pore size distribution. Some of these techniques are considered destructive, such as X-ray diffraction (XRD), where measurements are conducted on powdered samples. Others are micro-invasive, like laser-induced breakdown spectroscopy (LIBS), which involves creating a small hole (typically in the micrometer range) in the sample. There are also non-invasive techniques, including X-ray fluorescence (XRF), scanning electron microscopy (SEM), small-angle neutron scattering (SANS), and Fourier Transform Infrared absorption (FT-IR) spectroscopy [29,32,33,34,35,36]. An interesting and original approach is the use of coulometric analysis to correlate the water content with the firing temperatures of ceramic fragments [37]. Additionally, some studies have used colorimetric measurements to link changes in the raw material composition and reactions during firing to key characteristics of the fired products [31].

In this original work, the use of IBA techniques is proposed, more specifically the IBIL technique, to characterise the inorganic compounds present in the ceramic body of ancient Portuguese tiles (a set of 17 tiles) dated from the seventeenth to the twentieth centuries. The IBA data are complemented with colorimetry and X-ray diffraction (XRD) measurements, and the data are also obtained in a non-invasive mode. Moreover, the IBIL results will be correlated to the initial raw materials and manufacturing conditions of these ceramics, such as firing temperature and cooling down processes. PIXE, PIGE, and IBIL spectra can be collected simultaneously to provide both elemental and mineralogical information with a single sample analysis. This compositional characterisation could be an attractive and competitive alternative to other methods and techniques, most frequently used as described above, if sampling is not allowed or if the material amount is scarce.

## 2. Materials and Methods

The IBA experiments were performed under a He-rich atmosphere using an external 3.3 MeV proton beam produced by the Pelletron accelerator from the Physics Institute in UNAM (Mexico City, Mexico). The beam size is about 1 mm in diameter and the distance from the exit window to the sample is about 10 mm. Two X-ray detectors were used to record the low- and the high-energy X-ray signals coming from the sample. The low-energy X-ray detector, located at 135° from the beam direction and at a distance of 30 mm from the sample, is a Si-PIN high performance detector (XR-100CR, Bedford, MA, USA) with a 6 mm^2^ active area and a 0.5 μm thick Be window, allowing the detection of elements down to Na. High-energy X-rays were collected with a LEGe (Li) detector (Model GL0055P, Canberra-Mirion, Atlanta, GA, USA) with a 50 mm^2^ active area located at 135° from the beam direction and at a 45 mm distance from the sample. An 80 μm thick Al filter is added in front of this detector to attenuate the entrance of low-energy X-rays [20]. This LEGe detector also allows the simultaneous detection of gamma rays using a Princeton Gamma Tech amplifier (Princeton, NJ, USA). The light emitted was collected through a 74–vis lens collimator and a 600 μm diameter optical fibre connected to a USB2000 Ocean Optics Spectrometer (Orlando, FL, USA) placed at 45° to the beam direction and at a distance of 3 cm from the sample. More experimental details can be found elsewhere [19,24]. While IBIL spectra were recorded at 2.5 s, PIXE and PIGE spectra were recorded at 4 min on the same region. Concentrations from PIXE spectra were obtained using the software GUPIXWIN v 2.2.3. Standard sediment reference materials from NIST (SRM 2704 and SRM 2711) were also irradiated under the same conditions.

For colorimetric analysis, a portable colorimeter (NCS Colourpin, Stockholm, Sweden) with a spot size of 4 mm was used. It uses full spectrum white LEDs as an illuminant; the colour reflected by the sample simulates an observer at 2°, with an illuminant/observer angle of 45°. The colour is determined through the L*a*b* colour space parameters, according to the positive (or negative) values of the coordinates: positive a* values are for red colours (negatives are for green), positive values of b* are for yellow colours (blue for negative values), while the L* values (from 0 to 100) define lightness.

Additional X-ray diffraction measurements were performed using a D8-Bruker diffractometer without any surface preparations in order to avoid the use of powder and to preserve the integrity of the sample.

**Figure 1:**
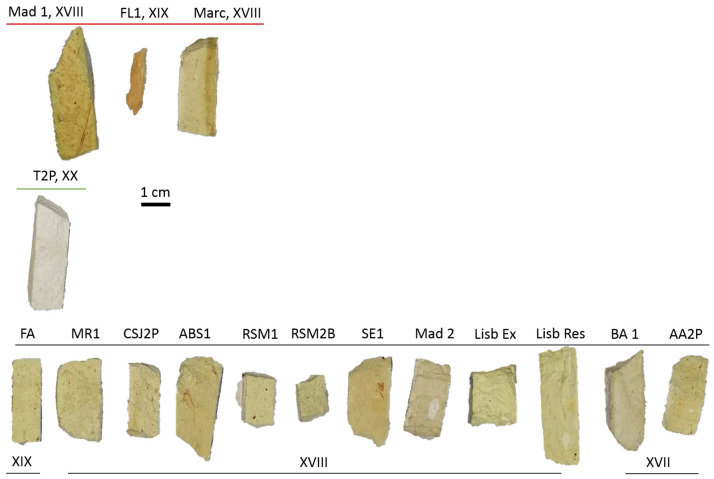
The ceramic body of the tiles analysed.

The set of analysed tiles (the ceramic body) was supplied by the Department of Cultural Heritage (DHC) of Lisbon City Hall, and the primary objective was to characterise them without causing any damage or requiring sampling. This set includes tiles from the mid-seventeenth century to the first half of the twentieth century, sourced from Lisbon and Arrábida, Portugal (see Figure 1). In Figure 1, each tile is labelled with its date and a code name that indicates its collection site. Additionally, the tiles in the figure are classified into three groups to facilitate the discussion in the manuscript.

Due to the lack of data available regarding the ionoluminescence emission bands in some of the minerals found during the study, cathodoluminescence (CL) data were considered as references when needed. This assumption is based on the fact that IBIL, as an analytical technique, is based on principles similar to those of CL, which is regularly used for the analysis of minerals and semiconductors [38]. This similarity allows the use of CL spectra databases for the analysis of IBIL features arising from different compounds [39]. It should be noted that available emission data are usually recorded from pure samples specifically prepared for measurement, while in this work, the materials under study are a mixture of minerals with minimal surface preparation. In this sense, this work also aims to address the lack of available IBIL data.

## 3. Results and Discussion

**Figure 2:**
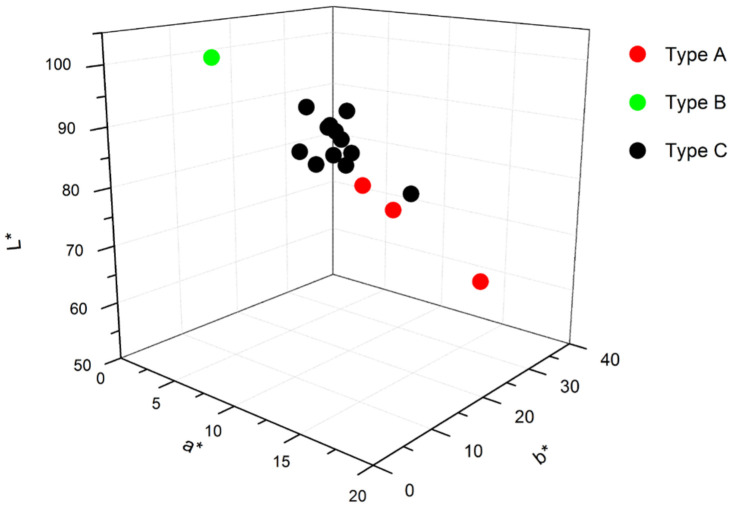
A 3D representation of the colorimetric coordinates of the ceramics analysed. The colour coding follows the scheme used throughout the manuscript; see text for details.

The ceramic bodies analysed in this work present a creamy colour (see Figure 1 and Figure 2), except for two of them. The darker one (reddish) corresponds to a tile from the eighteenth century (second half), denoted as FL1 in Figure 1. Despite its distinct appearance, no remarkable differences in elemental composition were found when compared with the rest of the tiles, as will be discussed later. On the other hand, the whitest ceramic (denoted as T2P in Figure 1), with a L* value very close to 100, is from the most recent tile (second quarter of the twentieth century).

**Figure 3:**
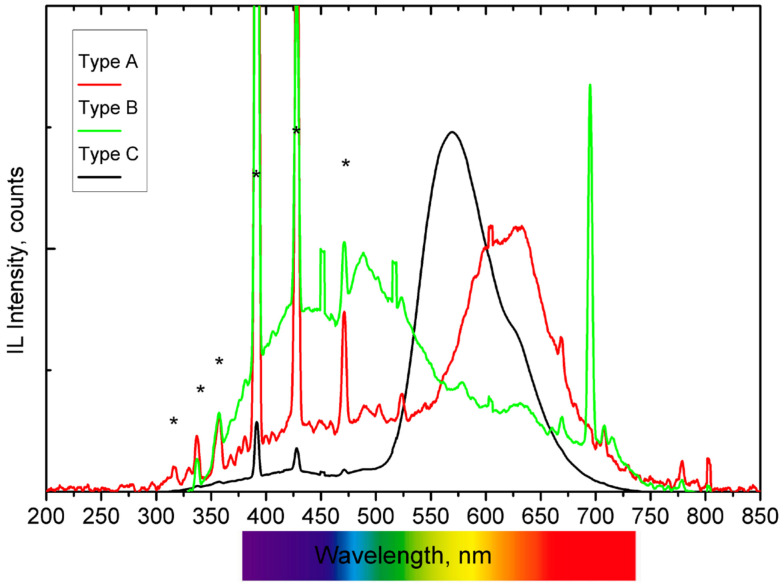
IBIL spectra of the ceramics (* indicates He emissions). Type A, from an eighteenth century ceramic tile, is characterised by a broad emission band centred around 630 nm. Type B, from a ceramic tile dating to the second quarter of the twentieth, is characterised by two broad emission bands and a sharp emission at approximately 690 nm. Type C, which includes most of the ceramic tiles, shows a band centred around 569 nm.

Representative IBIL spectra obtained during the analysis are presented in Figure 3, where the intense, sharp emissions attributed to the He emission (from the atmosphere) are highlighted. These He luminescence peaks can be used as a beam monitor as well as for the normalization of the luminescence spectra. Based on the wavelength of the maximum intensity of the light emitted when bombarded with the proton beam, the ceramics can be classified into three main groups: A, B, and C. Type A ceramics have one main broad band contribution centred in the orange-red region, with some sharp emissions throughout the studied region. Only one ceramic, corresponding to the most recent tile, exhibits a sharp emission at 695 nm and two broad emissions in the light and dark blue region (type B). Finally, most of the ceramics fall into the type C category, where the maximum emission intensity is found in the yellow region.

The IBIL spectrum classifies type A as a tile from the eighteenth century and is similar to those recorded for the other two tiles from the eighteenth and nineteenth centuries, all displaying a broad emission with a maximum centred at around 630 nm. Previous IBIL analyses have associated this emission with the presence of calcite and, more specifically, with the presence of Mn^2+^ impurities and their related transitions from the excited states ^4^F, ^4^D, ^4^P, and ^4^G to the lowest energy level ^6^S of the Mn^2+^ ion [40,41].

**Figure 4:**
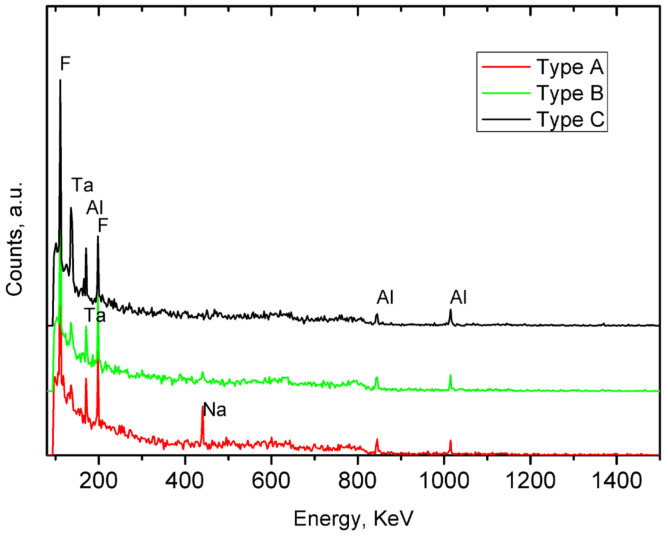
PIGE spectra recorded in the different types of ceramics with energy peaks marked for the relevant elements. The corresponding energies are as follows: F (110 and 197 keV), Al (171, 844 and 1014 keV), Na (440 keV), and Ta (136 and 165 keV). The presence of Ta is related to tantalum energy stabilizers in the beam line.

In these three ceramics, the presence of Na was identified at 440 keV by PIGE (see Table 1 and Figure 4). This is a distinguishing factor compared to the other samples, where Na was either not detected or present in very low concentrations. Sodium-rich fluxes, such as soda feldspar or sodium carbonate, were commonly used to lower the melting temperature during manufacturing. This could explain the presence of calcite in the type A samples, as the temperature reached inside the kiln was insufficient to decompose the carbonate minerals, which typically occurs between 800 and 950 °C. Additionally, PIXE analysis detected relatively high concentrations of Si in these samples (see Table 1). Considering the emission bands in the blue region of the IBIL spectrum, these can be associated with the presence of quartz [41].

The Type B IBIL spectrum was recorded from a ceramic tile dating to the second quarter of the twentieth century, the most recent and whitest tile analysed in this work. The spectrum’s main feature is a sharp emission at 695 nm, which is associated in the literature with the presence of Cr^3+^ ions (transitions from ^2^E_2_ to level ^4^A_2_) [38]. Although Cr was not identified by PIXE, its presence cannot be entirely ruled out, as IBIL is an extremely sensitive technique capable of detecting light emitters present at sub-ppm concentrations [24]. The spectrum also exhibits two bands in the blue region. Yang et al. [41] reported similar IBIL band structures in natural quartz samples, which were linked to the characteristic emissions of igneous quartz. Additionally, cristobalite and tridymite, high-temperature polymorphs of silica, have been reported to exhibit blue CL signals with an emission maximum at 450 nm [42]. According to the SiO_2_ phase diagram, these two polymorphs are stable only under low pressure and high temperature conditions (above 1000 °C for cristobalite and 1400 °C for tridymite) and become metastable if subjected to a fast cooling down process.

This tile differs in elemental composition (as shown by PIXE and PIGE data in Table 1), having the lowest Ca and the highest Si and Al concentrations of the set, indicating a different manufacturing method and starting materials. The results suggest that the tile was produced under high-temperature conditions followed by a fast cooling, with SiO_2_ as the primary compound of the mixture. Furthermore, the compositional data imply that the raw materials used for this tile were significantly different from those used for the other tiles, as reflected by the notably low levels of Ca and K, and the elevated levels of Si and Al.

The high-intensity Type C emission displays a band with the maximum located at 560 nm. This emission is associated with silicon dioxide and is attributed to the triplet luminescence of a twofold coordinate silicon or self-trapped excitons in an oxygen vacancy [43]. In this IBIL spectrum, which corresponds to a tile from the first quarter of the twentieth century, the 560 nm band shows a shoulder centred at 625 nm (similar to type A and the Mn^2+^-related emissions), indicating that the main constituent is SiO_2_ and a low concentration of calcite.

**Figure 5:**
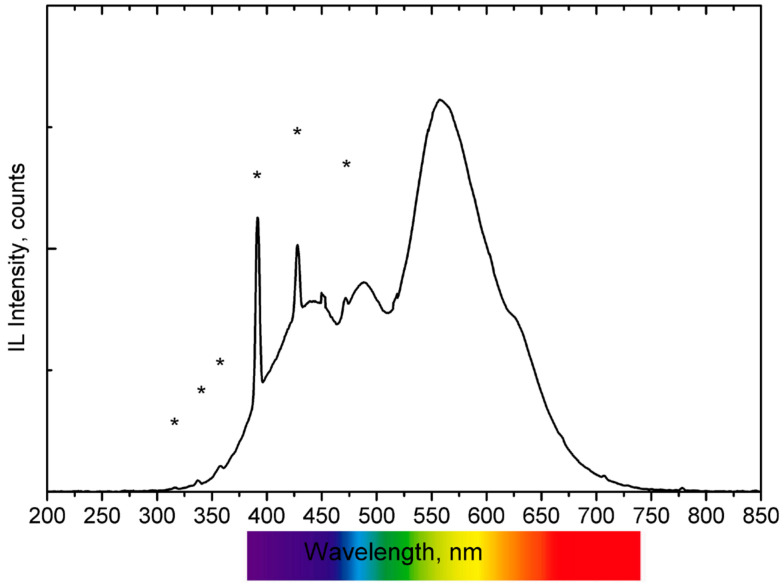
IBIL spectrum recorded in one of the ceramic bodies characterised (* indicates He emissions).

Most of the remaining tiles analysed exhibit IBIL spectra that can be described as a combination of the emissions seen in the previously described reference IBIL types, such as the one shown in Figure 5. These spectra feature a band centred at 560 nm, a shoulder at 625 nm (as in type C), and two additional emissions in the blue region (similar to type B). These results suggest that high temperatures were used in the kiln to decompose almost totally the initial carbonate minerals, followed by relatively slow annealing conditions that allowed for the transformation of nearly all silica polymorphs into fused silica.

**Figure 6:**
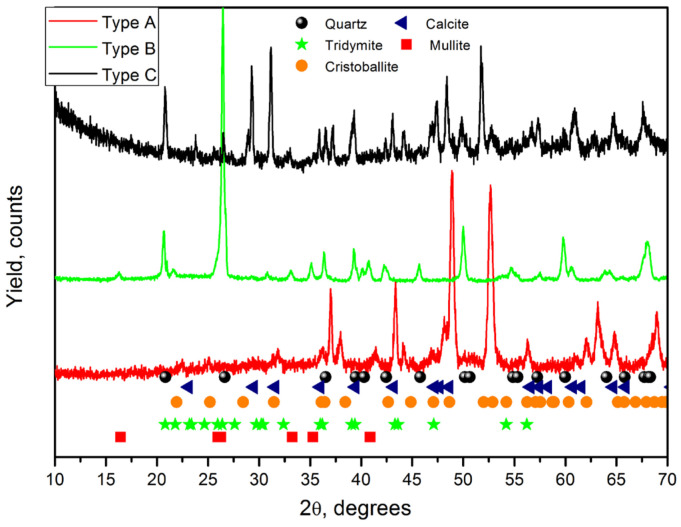
XRD diffractograms of selected tiles depending on the type of IL emission.

The results and conclusions obtained with IBIL data can be compared to the X-ray diffraction measurements performed on selected tiles from each group (see Figure 6). XRD diffractograms confirm the presence of calcite, fused silica, and, depending on the tile, polymorphs of silica. An XRD diffractogram for the type A ceramic shows signatures of calcite and quartz, indicting a relatively low firing temperature during manufacturing, as also concluded from IBIL data. On the other hand, the diffractograms for the other two types reveal a mixture of quartz and other compounds, including mullite, cristobalite, and tridymite, again suggesting the use of higher firing temperatures. The presence of mullite (Al_6_Si_2_O_13_), a high-temperature aluminium phase, can explain the elevated levels of Al and Si elements obtained in these samples (Table 1), further supporting the use of different raw materials.

Temperatures above 800 °C lead to shrinkage and reduced pore density in ceramics, which affects their permeability. Although lead is not among the starting materials of the paste, it was detected in almost all samples studied. The concentrations found (0.08–0.09 wt.%) do not indicate that it is an impurity from the raw materials; rather, it may be related to its diffusion from the glaze, where it is typically incorporated as PbO. Coroado et al. [44] attributed the presence of Pb in the ceramic body of tiles as a consequence of the capillarity effect. Lead can migrate into the porous body of the ceramic, moving through the material until it reaches the bottom. The fact that the most recent tile shows the lowest lead concentration suggests that it has lower porosity, indicating that higher temperatures and firing times were used during its manufacturing process. Porosity is a crucial factor that significantly influences the properties of ceramics, as it affects their mechanical strength, resistance to chemical erosion, and various other characteristics. The porosity of a ceramic material is primarily determined by the composition of the raw clay and the firing temperature used during production [29].

## 4. Conclusions

In conclusion, IBIL measurements, combined with the simultaneously obtained data from PIXE and PIGE techniques, allowed the identification of inorganic minerals present in Portuguese ceramic tiles from various centuries. The luminescence features provided fingerprints of key minerals, allowing for insights into the manufacturing processes, particularly firing temperature and cooling conditions. IBA and XRD data were compared, and the results demonstrate that IBA techniques, especially IBIL, are complementary valuable tools to easily characterise geological samples and cultural heritage objects, identifying the existing minerals and the overall inorganic composition while profiting from the non-destructive nature of the techniques and the ability to do the measurements in air without requiring sampling or conductive coatings.

## Figures and Tables

**Table 1:** Average chemical composition of ceramic bodies, obtained from PIXE (wt.%) for A, B and C types. “-”: below the detection limits; Na identification (not quantified) was completed by means of PIGE: D (detected), D * (detected with high standard variation and high uncertainties).

	Na	Al_2_O_3_	SiO_2_	P_2_O_5_	S	Cl	K_2_O	CaO	Cr_2_O_3_	Fe_2_O_3_	TiO_2_	MnO	SrO	PbO
**A**	D	36.70	51.21	1.04	0.047	1.08	1.09	7.53	0.01	1.17	0.01	0.01	0.01	0.09
**B**	-	43.52	55.56	0.28	-	0.02	0.43	0.08	-	0.10	0.01	-	-	-
**C**	D *	37.89	49.24	0.57	0.09	0.13	0.66	10.33	0.01	0.99	-	0.01	0.01	0.08

## Data Availability

Data are available under request.
